# Letter to the Editor with # CTM2‐2023‐10‐2488 entitled ‘Antibody responses as correlates of protection against SARS‐CoV‐2 in the Omicron era: A 5‐month prospective cohort study in Korean healthcare workers’

**DOI:** 10.1002/ctm2.1551

**Published:** 2024-01-27

**Authors:** So Yun Lim, Jineui Kim, Ji‐Soo Kwon, Sung‐Woon Kang, Seung‐Beom Kim, Woori Kim, Ju Yeon Son, Choi Young Jang, Heedo Park, Jeonghun Kim, Sohyun Lee, Kyung Taek Kim, Jaeuk Choi, Ji Yeun Kim, Joon Seo Lim, Euijin Chang, Seongman Bae, Jiwon Jung, Min Jae Kim, Yong Pil Chong, Sang‐Oh Lee, Sang‐Ho Choi, Yang Soo Kim, Man‐Seong Park, Sung‐Han Kim

**Affiliations:** ^1^ Department of Infectious Diseases Asan Medical Center University of Ulsan College of Medicine Seoul South Korea; ^2^ Division of Infectious Diseases, Department of Internal Medicine National Medical Center Seoul South Korea; ^3^ Department of Microbiology Institute for Viral Diseases, Vaccine Innovation Center College of Medicine, Korea University Seoul South Korea; ^4^ Clinical Research Center Asan Institute for Life Sciences, Asan Medical Center University of Ulsan College of Medicine Seoul South Korea

1

To the Editor:

Following the rollout of coronavirus disease‐19 (COVID‐19) vaccines, serum anti‐spike protein IgG antibodies and neutralising antibodies (nAb) against severe acute respiratory syndrome coronavirus 2 (SARS‐CoV‐2) have been accepted as correlates of protection.^1^, ^2^, ^3^ Meanwhile, considering that these correlates of protection were determined based on studies involving individuals who had not previously been infected, it is crucial to re‐evaluate the correlates of protection within the context of the complex immunity of the population established during the Omicron wave that led a significant portion of the population to acquire hybrid immunity. Hybrid immunity, which is achieved through exposure to both natural SARS‐CoV‐2 infection and the vaccine, has been found to offer more long‐lasting and comprehensive protection against COVID‐19 compared to vaccination or infection alone.^4^ Consistent with this, robust epidemiological data suggest that hybrid immunity provides substantial protection against subsequent infection,^5^ particularly in the context of the Omicron variant.^6^, ^7^ Therefore, we aimed to investigate the immune correlates of protection against COVID‐19 in populations with complex immune statuses in the Omicron era.

A cohort of healthcare workers was enrolled at Asan Medical Center, the largest tertiary medical centre in Seoul, South Korea, with 2700 beds, between December 2022 and January 2023, who had completed a booster vaccination and had either planned to receive the bivalent booster vaccination or not. Blood and saliva samples were collected, and ELISA and virus neutralisation tests were used for immunological assessments. The titres of SARS‐CoV‐2 S1‐specific serum IgG antibody and saliva IgA antibody were determined using an in‐house ELISA that utilised the Wuhan‐Hu‐1 and Omicron BA.5 subvariant spike protein S1 as antigens. The 50% neutralising dose (ND50) against BA.5 was determined using the virus reduction neutralisation test (VRNT). The detailed definition, immunological evaluation and statistical analysis are described in the Supporting Information.

A total of 482 participants were enrolled in this study (Figure S3), and 69 individuals (14.3%) experienced subsequent SARS‐CoV‐2 infection during the 5‐month observation period. Demographic and baseline characteristics of the study participants, categorised by subsequent infection, are presented in Table S2. Participants who had a subsequent SARS‐CoV‐2 infection had significantly lower baseline serum antibody levels of Wuhan S1‐IgG, BA.5 S1‐IgG and nAb against BA.5 compared to those who did not experience a subsequent infection (all *p* < .001) (Figure 1A–C). However, baseline saliva levels of Wuhan S1‐IgA (*p* = .42) and BA.5 S1‐IgA (*p* = .18) antibodies did not show significant differences between individuals who experienced subsequent infection and those who did not (Figure 1D,E). We analysed the distribution of participants with subsequent infection based on their antibody levels. The infection rate showed significant decreasing trends as serum antibody levels increased (Figure 2A–C, all *p* for trend <.01), while no significant trend was observed with saliva antibody levels (Figure 2D,E). With the optimal cutoff values determined from the receiver operating characteristic curves (Figure S4), multivariable Cox regression analysis showed that hybrid immunity (adjusted hazard ratio [aHR] 0.20 in analysis with Wuhan S1‐IgG, aHR 0.20 with BA.5 S1‐IgG; aHR 0.19 with nAb against BA.5; all *p* < .01) and baseline serum antibody level higher than cutoff value (aHR 0.43 for Wuhan S1‐IgG, *p =* .02; aHR 0.32 for BA.5 S1‐IgG, *p* = .005; aHR 0.26 for nAb against BA.5, *p* = .045) were independent protective factors against subsequent SARS‐CoV‐2 infection during the 5‐month follow‐up period; in contrast, saliva IgA antibody levels did not show a protective effect (Table 1). Additionally, Wuhan S1‐IgG showed the highest sensitivity (72.9%) compared with nAb against BA.5 (64.3%) and BA.5 S1‐IgG (62.7%). Moreover, combining hybrid immunity with antibody levels increased the sensitivity of Wuhan S1‐IgG to 89.0%, BA.5 S1‐IgG to 88.5% and nAb against BA.5 to 87.2%, respectively (Table 2).

**FIGURE 1:**
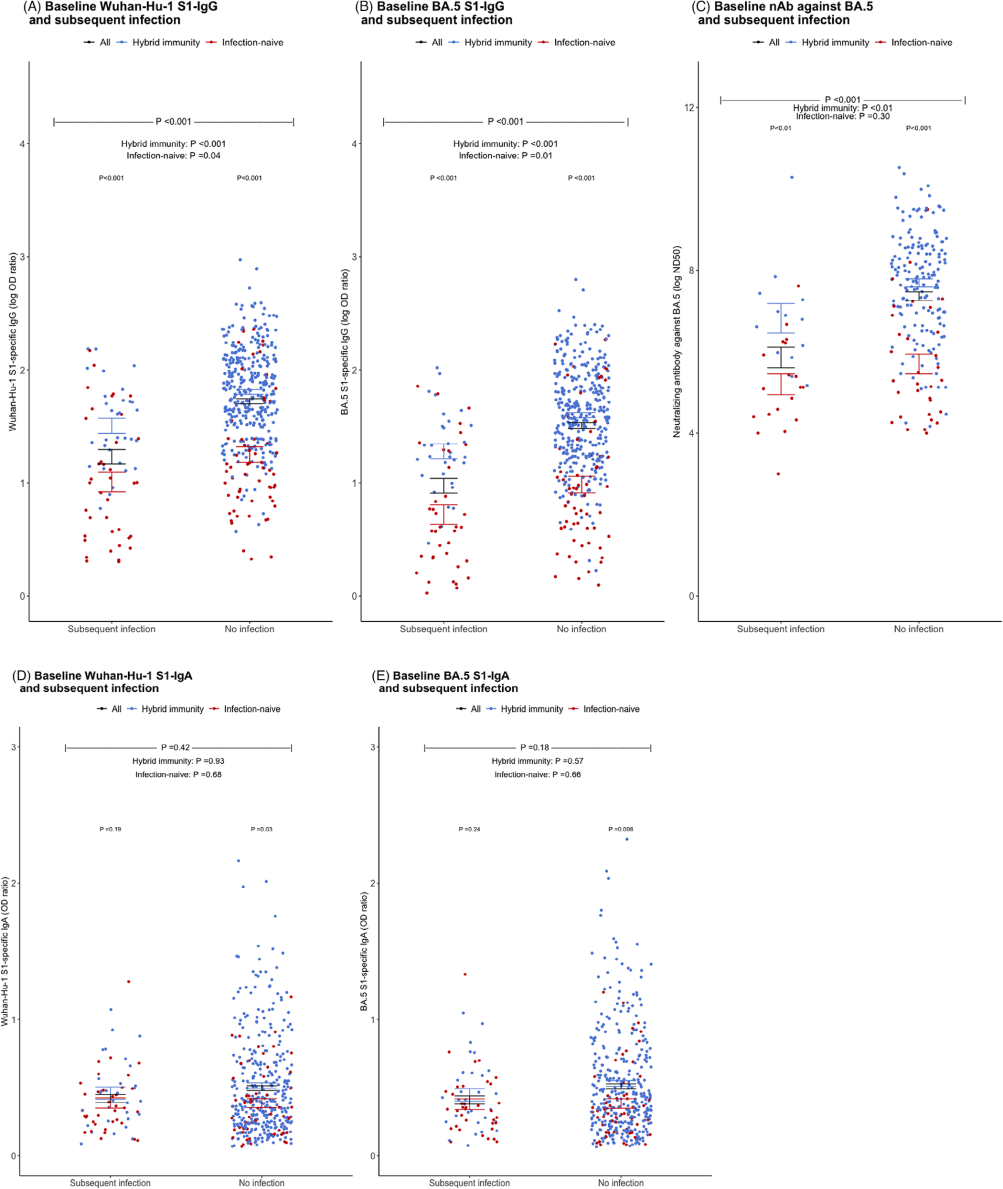
Baseline serum and saliva antibody levels according to the subsequent SARS‐CoV‐2 infection. (A) Serum Wuhan‐Hu‐1 S1‐specific IgG. (B) Serum BA.5 S1‐specific IgG. (C) Serum neutralising antibody against BA.5. (D) Saliva Wuhan‐Hu‐1 S1‐specific IgA. (E) Saliva BA.5 S1‐specific IgA. Horizontal lines indicate mean ± standard error.

FIGURE 2Participants with subsequent infection stratified by baseline SARS‐CoV‐2 antibody levels. (A) By serum Wuhan‐Hu‐1 S1‐IgG. (B) By serum BA.5 S1‐IgG. (C) By serum neutralising antibody against BA.5. (D) By saliva Wuhan‐Hu‐1 S1‐IgA. (E) By saliva BA.5 S1‐IgA. Blue bars represent the distribution of infected individuals attributed to each specific antibody titre. Red dots indicate the infection rate at each specific antibody titre. Black lines indicate the probability of getting infected with antibody titres exceeding the given values, providing cumulative incidence, and the grey shaded area denotes the 95% confidential interval. The *p*‐value for the trend of the infection rate was calculated using the Cochran–Armitage test. The cutoff levels corresponding to 50% and 80% of the infected individuals were indicated for the serum antibody responses. The *x*‐axis of saliva antibody levels was divided into quartiles.

**Figure:**
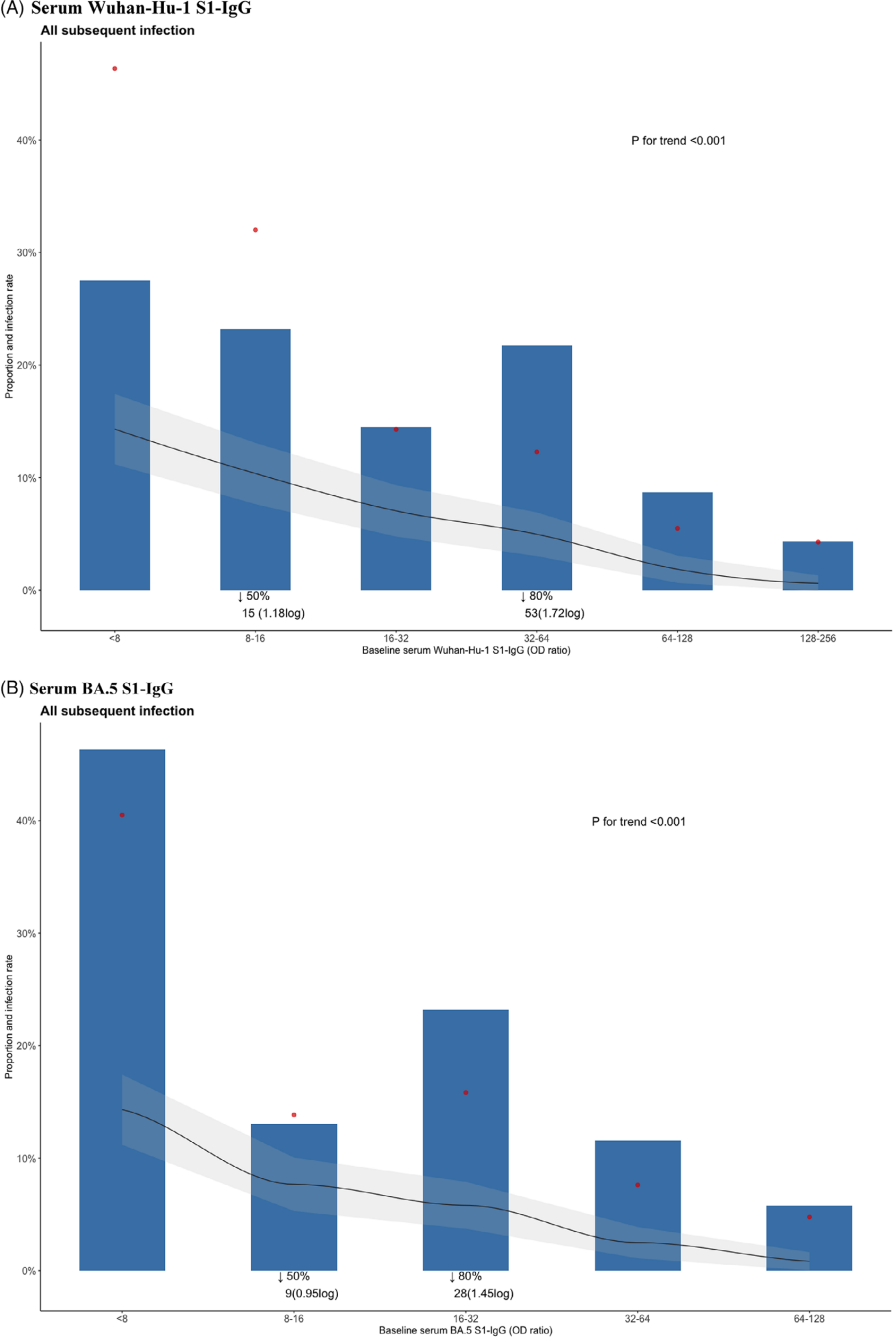

**Figure:**
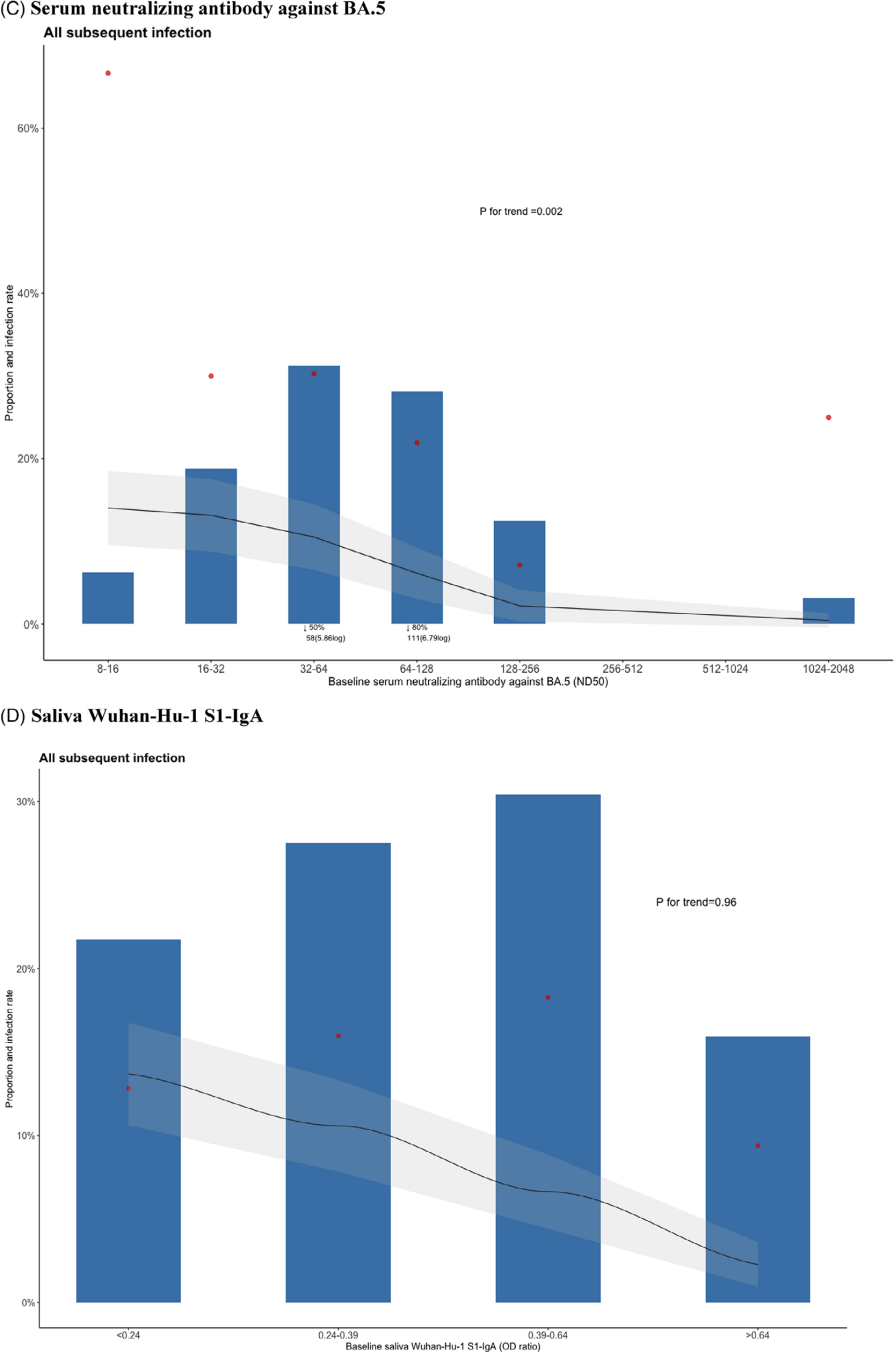

**Figure:**
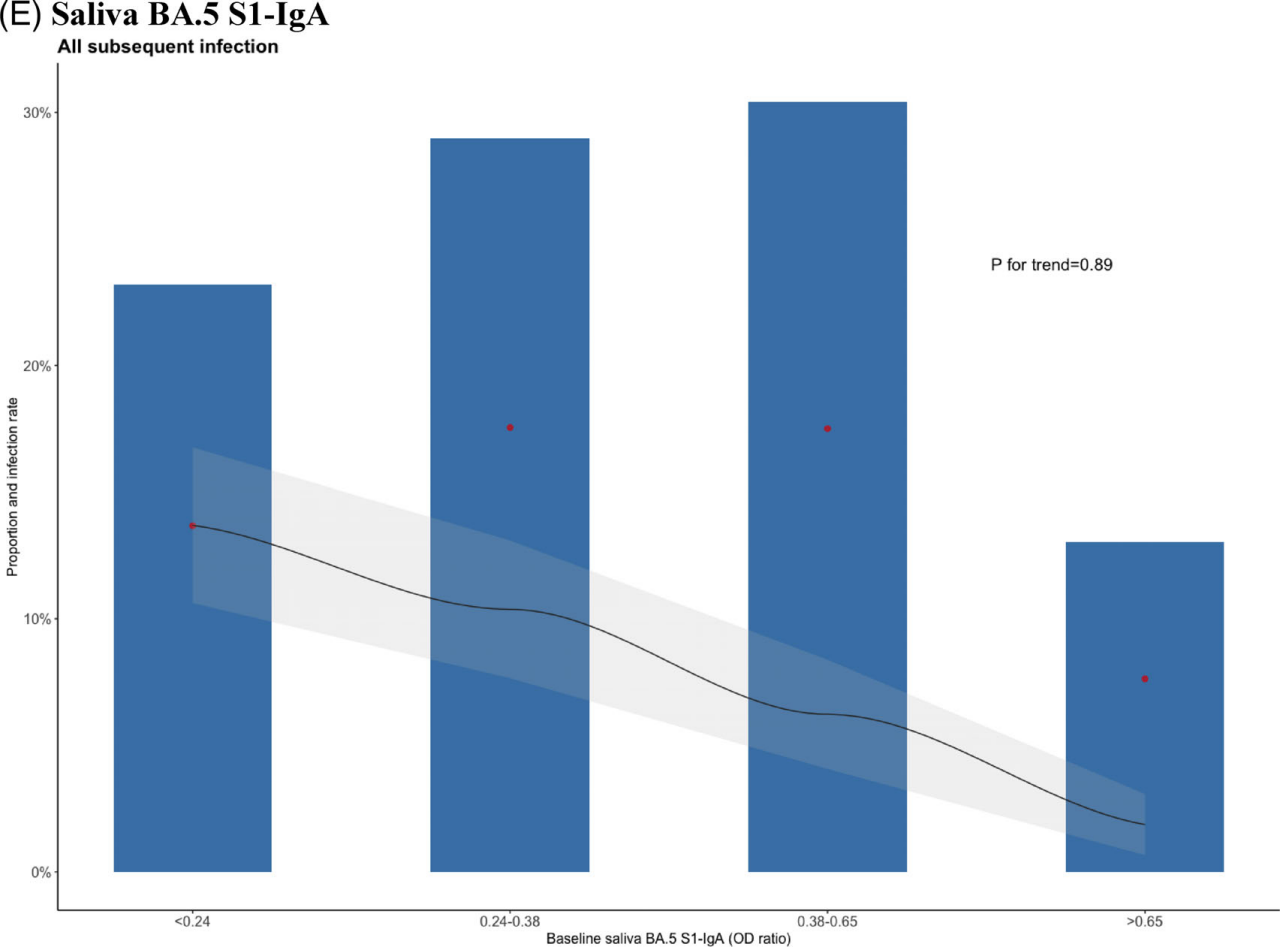

**TABLE 1:** Cox proportional hazard model for prediction of subsequent infection using baseline antibody levels.

	Univariate analysis		Multivariable analysis	
Variable (prediction by baseline serum Wuhan S1‐IgG) (*n* = 466)	Hazard ratio (95% confidence interval)	*p*‐Value	Adjusted hazard ratio (95% confidence interval)	*p*‐Value
Homologous previous vaccination	1.30 [0.67−2.53]	.44		
Hybrid immunity	0.13 [0.07−0.23]	<.001	0.20 [0.10−0.39]	<.001
Bivalent BA.4/5 booster vaccination	1.40 [0.81−2.41]	.23		
Time since last exposure to SARS‐CoV‐2 antigen^a^ before study enrollment (≤90 days)	0.16 [0.02−1.17]	.07	0.46 [0.06−3.53]	.46
Baseline serum Wuhan S1‐specific IgG ≥ 1.36^b^	0.20 [0.11−0.35]	<.001	0.43 [0.21−0.87]	.02
**Variable (prediction by baseline serum BA.5 S1‐IgG)** **(*n* = 466)**				
Homologous previous vaccination	1.30 [0.67−2.53]	.44		
Hybrid immunity	0.13 [0.07−0.23]	<.001	0.20 [0.10−0.37]	<.001
Bivalent BA.4/5 booster vaccination	1.40 [0.81−2.41]	.23		
Time since last exposure to SARS‐CoV‐2 antigen^a^ before study enrollment (≤90 days)	0.16 [0.02−1.17]	.07	0.55 [0.07−4.25]	.57
Baseline serum BA.5 S1‐specific IgG level ≥1.43^b^	0.15 [0.08−0.31]	<.001	0.32 [0.15−0.71]	.005
**Variable (prediction by baseline serum neutralising antibody against BA.5)** **(*n* = 219)**				
Homologous previous vaccination	1.30 [0.67−2.53]	.44		
Hybrid immunity	0.13 [0.07−0.23]	<.001	0.19 [0.07−0.51]	.001
Bivalent BA.4/5 booster vaccination	1.40 [0.81−2.41]	.23		
Time since last exposure to SARS‐CoV‐2 antigen^a^ before study enrollment (≤90 days)	0.16 [0.02−1.17]	.07	0.00 [0.00−NA]	>.99
Baseline serum neutralising antibody against BA.5 ≥7.00^b^	0.09 [0.03−0.32]	<.001	0.26 [0.07−0.97]	.045
**Variable (prediction by baseline saliva Wuhan S1‐IgA)** **(*n* = 454)**				
Homologous previous vaccination	1.30 [0.67−2.53]	.44		
Hybrid immunity	0.13 [0.07−0.23]	<.001	0.14 [0.08−0.26]	<.001
Bivalent BA.4/5 booster vaccination	1.40 [0.81−2.41]	.23		
Time since last exposure to SARS‐CoV‐2 antigen^a^ before study enrollment (≤90 days)	0.16 [0.02−1.17]	.07	0.43 [0.06−3.26]	.42
Baseline saliva Wuhan S1‐specific IgA ≥0.53^b^	0.49 [0.25−0.99]	.045	0.73 [0.36−1.47]	.38
**Variable (prediction by baseline saliva BA.5 S1‐IgA)** **(*n* = 454)**				
Homologous previous vaccination	1.30 [0.67−2.53]	.44		
Hybrid immunity	0.13 [0.07−0.23]	<.001	0.14 [0.08−0.27]	<.001
Bivalent BA.4/5 booster vaccination	1.40 [0.81−2.41]	.23		
Time since last exposure to SARS‐CoV‐2 antigen^a^ before study enrollment (≤90 days)	0.16 [0.02−1.17]	.07	0.45 [0.06−3.36]	.43
Baseline saliva BA.5 S1‐specific IgA level ≥0.76^b^	0.25 [0.08−0.81]	.02	0.40 [0.12−1.29]	.12

Abbreviation: NA, not available.

^a^ Either vaccination or infection.

^b^ Optimal cutoff value determined from ROC curve (values are presented as the log_10_ of the OD ratio for IgG and log_2_ of the ND50 for neutralising antibody).

**TABLE 2:** Predictive performance of baseline serum Wuhan S1‐IgG, BA.5 S1‐IgG and nAb against BA.5 at optimal cutoff values for protection against subsequent SARS‐CoV‐2 infection.

	Sensitivity (%) (*n*/*N*, 95% CI)	Specificity (%) (*n*/*N*, 95% CI)	PPV (%) (95% CI)	NPV (%) (95% CI)
**Baseline Wuhan S1‐IgG** ^a^	72.9 (299/410, 68.4−77.2)	65.2 (45/69, 52.8−76.3)	92.6 (89.2−95.2)	28.7 (21.8−36.5)
**Baseline BA.5 S1‐IgG** ^a^	62.7 (257/410, 57.8−67.4)	78.3 (54/69, 66.7−87.3)	94.5 (91.1−96.9)	26.0 (20.1−32.5)
**Baseline nAb against BA.5** ^a^	64.3 (126/196, 57.2−71.0)	84.4 (27/32, 67.2−94.7)	96.1 (91.2−98.7)	28.3 (19.6−38.4)
**Hybrid immunity**	84.8 (350/413, 80.9−88.1)	55.1 (38/69, 42.6−67.1)	91.9 (88.7−94.4)	37.6 (28.2−47.8)
**Baseline Wuhan S1‐IgG + hybrid immunity**	89.0 (365/410, 85.6−91.9)	42.0 (29/69, 30.2−54.5)	90.2 (86.9−92.9)	39.0 (27.9−51.0)
**Baseline BA.5 S1‐IgG + hybrid immunity**	88.5 (363/410, 85.1−91.5)	47.8 (33/69, 35.7−60.2)	91.0 (87.8−93.7)	41.1 (30.2−52.7)
**Baseline nAb S1‐IgG + hybrid immunity**	87.2 (171/196, 81.8−91.6)	56.3 (18/32, 37.7−73.6)	92.3 (87.4−95.7)	42.4 (27.5−58.4)

Abbreviations: nAb, neutralising antibody; NPV, negative predictive value; PPV, positive predictive value; Wuhan, Wuhan‐Hu‐1.

^a^ Cutoff value: Wuhan S1‐IgG: 1.43 log OD ratio; BA.5 S1‐IgG: 1.36 log OD ratio; nAb against BA.5: 7.00 log_2_ ND50.

In this prospective cohort study with a 5‐month follow‐up, we have reaffirmed that antibody responses continue to serve as immunologic correlates of protection against COVID‐19 during the predominance of the Omicron variant and in populations with hybrid immunity. One recent study reported the association between baseline antibody levels before the fourth dose of the COVID‐19 vaccine and the rate of subsequent SARS‐CoV‐2 infection^8^; however, this study only included infection‐naïve individuals, and all participants received the booster vaccine. Thus, the effect of hybrid immunity and booster vaccination could not be considered in that study. In our study, nearly all participants with hybrid immunity had previous exposure to the Omicron variant (except for the unknown variant), and the study cohort included both, those who received the bivalent vaccine and those who did not. Therefore, we could collectively conduct a comprehensive analysis of immunologic correlates of protection against subsequent SARS‐CoV‐2 infection, which reflects the effect of hybrid immunity and booster vaccination in the Omicron era.

Also, hybrid immunity emerged as an independent protective factor against subsequent infections in this study. Hybrid immunity exposes the immune system not only to spike‐derived epitopes provided by COVID‐19 vaccines but also to a wide range of viral epitopes stimulated by natural SARS‐CoV‐2 infection, resulting in an enhanced immune response that targets both spike and non‐spike epitopes. Moreover, individuals with hybrid immunity acquired from natural SARS‐CoV‐2 infection have been found to develop mucosal immunity in the nasal cavity and tissue‐specific immunity (specifically lung) exclusively detected in individuals with hybrid immunity,^9^ and might contribute to protection against initial acquisition of SARS‐CoV‐2 Omicron infection.^10^ However, the baseline saliva IgA antibody in this study did not demonstrate a protective role: this may be attributed to predominantly mild past infections among participants, leading to insufficient development of mucosal immunity, and the substantial time elapsed between the previous infection and study enrollment, which might have caused a decrease in mucosal IgA levels. Also, given that the correlation between blood and saliva biomarkers may not be prominent,^11^ cautious interpretation is needed for biomarkers measured in the saliva. Further immunologic studies are needed to determine the exact mechanism.

In conclusion, this 5‐month observational cohort study showed that serum antibody responses, including serum‐binding antibodies and nAb, serve as immunological correlates of protection against subsequent SARS‐CoV‐2 infection, whereas saliva IgA does not. Also, hybrid immunity was identified as an independent protective factor against subsequent SARS‐CoV‐2 infection. High baseline serum antibody levels exceeding optimal cutoff levels, in combination with hybrid immunity, demonstrated a significant predictive performance for protecting against subsequent SARS‐CoV‐2 infection.

## AUTHOR CONTRIBUTIONS

So Yun Lim, Man‐Seong and Sung‐Han Kim conceived the study. Ji‐Soo Kwon, Sung‐Woon Kang, Seung‐Beom Kim, Woori Kim, Ju Yeon Son and Choi Young Jang contributed to the data curation and investigation. So Yun Lim, Jineui Kim, Ji‐Soo Kwon, Heedo Park, Jeonghun Kim, Sohyun Lee, Kyung Taek Kim, Jaeuk Choi and Ji Yeun Kim did the formal analysis and verified the data. Sohyun Lee was responsible for data visualisation. Sohyun Lee, Jineui Kim and Ji‐Soo Kwon wrote the original manuscript draft. Joon Seo Lim, Euijin Chang, Seongman Bae, Jiwon Jung, Min Jae Kim, Yong Pil Chong, Sang‐Oh Lee, Sang‐Ho Choi, Yang Soo Kim, Man‐Seong and Sung‐Han Kim reviewed and edited the manuscript. Man‐Seong and Sung‐Han Kim supervised the project and obtained the necessary funding to carry out the research.

## CONFLICT OF INTEREST STATEMENT

The authors declare they have no conflicts of interest.

## ETHICS STATEMENT

This study was approved by the institutional review board of Asan Medical Center (IRB No. 2022‐1269). All procedures were in accordance with the Declaration of Helsinki, and all participants gave informed consent.

## Supporting information

Supporting Information

## Data Availability

The data that support the findings of this study are available on request from the corresponding author. The data are not publicly available due to privacy or ethical restrictions.
