# Supplementary material for: Letter to the Editor with # CTM2‐2023‐10‐2488 entitled ‘Antibody responses as correlates of protection against SARS‐CoV‐2 in the Omicron era: A 5‐month prospective cohort study in Korean healthcare workers’
Source: Clin Transl Med. 2024 Jan 27;14(1):e1551. doi: 10.1002/ctm2.1551 (PMC10819075; doi:10.1002/ctm2.1551)
Supplement: Supplementary file 1 — Supporting Information [file CTM2-14-e1551-s001.docx]

**SUPPORTING INFORMATION**

**TABLE OF CONTENTS**

| **Detailed Methods** ............................................................................................................................................................................................... 2 |
| --- |
| **Summary of South Korea's COVID-19 vaccination and epidemic trends over time** ............................................................................... 2 |
| **Definitions**........................................................................................................................................................................................................ 2 |
| **Estimation of SARS-CoV-2 variants of previous infections**........................................................................................................................ 3 |
| **Immunological evaluation** ............................................................................................................................................................................. 4 |
| **Statistical analysis** ........................................................................................................................................................................................... 6 |
| **Extended Results** ................................................................................................................................................................................................ 7 |
| **Additional Discussion** .........................................................................................................................................................................................8 |
| **Study limitations** ................................................................................................................................................................................................. 9 |
| **Supplementary Tables** ......................................................................................................................................................................................10 |
| **Supplementary Figures** .................................................................................................................................................................................... 14 |
| **References** ......................................................................................................................................................................................................... 25 |

**DETAILED METHODS**

**Summary of South Korea's COVID-19 vaccination and epidemic trends over time**

South Korea introduced its COVID-19 vaccines in February 2021, including the AZD1222 (AstraZeneca, Cambridge, UK) and BNT162b2 (Pfizer-BioNTech, New York City, NY, USA, and Mainz, Germany). In the hospital setting, BNT162b2 was assigned to healthcare workers who were in close contact with COVID-19 patients and AZD1222 to those not directly involved with COVID-19 patient care in accordance with the South Korean government policy. Booster vaccination was conducted using mRNA vaccines in the Fall of 2021. After the booster vaccination, there have been three waves of COVID-19 caused by the Omicron SARS-CoV-2 variant in South Korea: the first wave from February to May 2022 was dominated by BA.1 subvariant at the beginning, but after a few weeks, BA.2 subvariant predominated, followed by BA.5 subvariant from July to October 2022, and finally BA.5 again from November 2022 to January 2023 with a slow overlap of the BN.1 subvariant (Figure S1)^1^. A substantial number of healthcare workers in South Korea who previously received booster vaccination and remained naïve to infection acquired hybrid immunity against COVID-19 during this Omicron wave (Figure S1).^1^

**Definitions**

Demographic characteristics, underlying diseases, medications, previous SARS-CoV-2 infection, and COVID-19 vaccination history were reviewed. Hybrid immunity was defined as a combination of a 3- or 4-dose COVID-19 vaccine and previous SARS-CoV-2 infection, which was identified as having a positive nasopharyngeal polymerase chain reaction (PCR) prior to the study as well as a positive nucleocapsid protein (NP) antibody detected in the baseline sampling of the participants. Individuals with a history of SARS-CoV-2 infection two or more times were excluded to ensure clarity in evaluating the concept of hybrid immunity. The type of previous vaccination was classified as heterologous and homologous vaccination: the former involved one or more doses of AZD1222 followed by mRNA vaccines, while the latter consisted of consecutive mRNA vaccinations. The BA.4/5 bivalent vaccine that the study participants received was all BNT162b5 (Pfizer-BioNTech, New York City, NY, USA, and Mainz, Germany). Subsequent infection was defined as an infection with SARS-CoV-2 within the 5 months following study enrollment. First, we instructed the participants to report any infections confirmed by SARS-CoV-2 PCR or rapid antigen test for SARS-CoV-2 infection that occurred after their study enrollment, and we conducted regular investigations for subsequent infection of the study participants. Second, to find asymptomatic cases of infection, we also conducted paired serologic tests of the SARS-CoV-2 nucleocapsid protein antibody. For infection-naïve individuals at the time of enrollment, those whose follow-up nucleocapsid protein antibody changed from negative to positive were presumed to have experienced a subsequent infection even if they did not self-report any infection. Lastly, as subsequent infections of participants with previous infections at the start of the study could not be determined based on seroconversion of the nucleocapsid protein antibody, asymptomatic infection in these individuals was defined as more than a two-fold increase of SARS-CoV-2 Wuhan-Hu-1 S1-IgG antibody titer during study period^2,3^ in those who did not receive a bivalent booster vaccination. The detailed information on subsequent infection is described in Table S1. This study was approved by the institutional review board of Asan Medical Center (IRB No. 2022-1269). All procedures were in accordance with the Declaration of Helsinki, and all participants were given informed consent.

**Estimation of SARS-CoV-2 variants of previous infections**

The SARS-CoV-2 variants from previous infections in individuals with hybrid immunity were estimated by taking into account the most common variants at the time of their infection. This information was obtained from the Korea Disease Control and Prevention Agency, covering the period from January 20, 2020, to the present.^4^

**Immunological evaluation**

The titers of SARS-CoV-2 S1-specific serum IgG antibody and saliva IgA antibody were determined using an in-house ELISA that utilized the Wuhan-Hu-1 and Omicron BA.5 subvariant spike protein S1 as antigens. Briefly, 2 ug/mL of recombinant SARS-CoV-2 Wuhan-Hu-1 or Omicron BA.5 subvariant protein (SinoBiological, Beijing, China) was coated onto 96-well plate (MaxiSorp; Thermo Fisher Scientific, Waltham, MA) overnight at 4 ºC, and then the plates were blocked with 1% bovine serum albumin in phosphate buffered saline (PBS). Serum samples diluted at 1/100 and extending to 1/1,000, 1/10,000, and beyond for IgG measurement, and saliva samples diluted at 1:50 or 1:500 for IgA measurement were added and incubated for 2 hours at room temperature. Saliva was self-collected by spitting in 50 mL conical tube. After collection, saliva samples were stored at −80 ºC until analyzed. Horseradish peroxidase-conjugated anti-human IgA (Jackson Immunoresearch, West Grove, PA) was used as secondary antibody. The plates were developed with 3,3’,5,5’-tetramethlbenzidine substrate (Sigma-Aldrich, St. Louis, MO, USA) and the reaction stopped with a stop solution (Sigma-Aldrich). Optical density (OD) was measured using a SpectraMax microplate reader (Molecular Devices LLC, San Jose, CA) at 450 nm. We standardized the in-house ELISA for S1-specific serum IgG using reference pooled sera from the International Vaccine Institute in Seoul, South Korea. However, for the BA.5 variant, there was no reference pool available. Therefore, antibody units were not normalized to international units and were measured as optical density (OD) at a wavelength of 450 nm for the purpose of statistical comparison. Wuhan-Hu-1 S1 and BA.5 S1-specific IgA titers were not normalized to international units globally; therefore, those titers were presented as relative OD values based on a 1:50 dilution factor.

To determine the cut-off values for the ELISA, the mean and standard deviation (SD) values of OD from negative control plasma that had not been exposed to SARS-CoV-2 were measured. The cut-off values were determined by calculating the mean OD plus three folds of the SD values, which was 0.4 for IgG as reported in previous studies5,6. To determine the cutoff values IgA ELISA, we measured the mean OD values from 15 negative control saliva samples from volunteers with negative SARS-CoV-2 PCR results and 11 positive control saliva samples from COVID-19 patients. The cutoff value was OD value 0.75, determined by calculating the mean OD of negative plus 0.13-fold of the mean OD of positive, as described in previous study7.

The diagnostic performance of Wuhan-Hu-1 S1 IgG ELISA with plasma samples from COVID-19 patients and unexposed individuals, sensitivity was 100% (95% CI 85.2-100) and specificity was 100% (95% CI 89.1-100). The in-house ELISA for S1-specific saliva IgA and the commercially available SARS-CoV-2 IgA ELISA kit (EuroImmun, Lübeck, Germany) showed a high correlation coefficient of 0.74 (*p*<0.001), with a 100% (95% CI 83.2–100) sensitivity and 100% (95% CI 85.8–100) specificity when the commercial kit was used as the standard.

The 50% neutralizing dose (ND50) was determined using the virus reduction neutralization test (VRNT).^8^ Briefly, the BA.5 variants of SARS-CoV-2 were directly detected with fluorescent antibodies in a 96-well plate, and the nAb titers were determined by analyzing the number of infected cells. There was a strong correlation (Pearson r = 0.96; *p*<0.001) between the results of the plaque reduction neutralization test (PRNT) and VRNT in the laboratory. Also, both Wuhan-S1 IgG and BA.5 S1-IgG showed a good correlation with the VRNT assay (Spearman ρ=0.73 and ρ=0.80, respectively; both *p*<0.001) (Figure S2).

**Statistical analysis**

The titer of the S1-specific IgG and IgA antibodies was log-transformed using base 10, and the ND50 was transformed using base 2. The χ^2^ or Fisher's exact test was used to compare categorical variables, while the Wilcoxon rank-sum test was used to compare continuous variables, as appropriate. The correlation between the two variables was analyzed using Pearson correlation analysis or Spearman's rank correlation analysis, as appropriate. The *p* value for trend was calculated using the Cochran-Armitage test. The area under the receiver operating characteristic (AUROC) curve was calculated for each immune marker to measure its ability to discriminate subsequent SARS-CoV-2 infection. The DeLong test was used to compare the ROC curves. A cutoff value for each immune marker was determined using the Youden index.^9^ The sensitivity, specificity, positive predictive value, and negative predictive value, along with their corresponding 95% confidence intervals (CI), were calculated using the optimal cutoff values for each immune marker to evaluate the predictive performance for protection against subsequent SARS-CoV-2 infection. A Cox proportional hazard model was used to estimate the protective factors against subsequent SARS-CoV-2 infection after study enrollment. Variables with *p* values <.10 in the univariate analysis were included in the multivariable analysis. Kaplan-Meier methods were used to plot cumulative incidence curves for subsequent infections. All tests of significance were two-tailed, and *p* value <.05 were considered to indicate statistical significance. Data analysis and graph plotting were conducted using R software version 4.1.3 (R Project for Statistical Computing, Vienna, Austria).

**EXTENDED RESULTS**

**Subgroup analysis 1-month after bivalent vaccine administration**

Subgroup analysis was also conducted to assess the impact of the bivalent vaccine on antibody responses after excluding participants who were infected within 1 month after vaccination. A total of 470 participants were included in the subgroup analysis, with 57 participants (12.1%) who experienced subsequent infection.

In the 1-month subgroup analysis, the participants who received the bivalent vaccine were assessed based on their antibody levels 1 month after vaccination, while those who did not receive the bivalent vaccine were evaluated using their baseline antibody levels. The optimal cutoff values for distinguishing subsequent infection in the 1-month subgroup analysis were determined to be 1.85 log OD ratio for serum Wuhan-Hu-1 S1-IgG, 1.55 log OD ratio for serum BA.5 S1-IgG, and 7.86 log_2_ND50 for serum nAb against BA.5, respectively (Figure S5).

Hybrid immunity and antibody levels exceeding the optimal cutoff value were also significant protective factors against subsequent infection in the 1-month subgroup analysis (Table S3). However, there was no significant difference in the cumulative incidence of subsequent infection based on the administration of the bivalent vaccine in the 1-month subgroup analysis (Figure S6). Differences in clinical characteristics based on the administration of the bivalent vaccine are detailed in Table S4, while antibody levels pre- and post-administration of the bivalent vaccine are shown in Figure S7.

**ADDITIONAL DISCUSSION**

Interestingly, the participants who did not receive the bivalent vaccine had a significantly higher proportion of previous SARS-CoV-2 infection and significantly higher baseline antibody levels compared to those who received the bivalent vaccine. Additionally, the individuals who received the bivalent vaccine were significantly older than those who did not (Table S4). Considering these unfavorable conditions for SARS-CoV-2 infection among recipients of the bivalent vaccine, it is plausible that these factors might have contributed to the lack of protective effect of bivalent vaccination in the cumulative incidence of subsequent infection following the administration of the bivalent booster vaccine. Therefore, our finding that bivalent vaccination was not associated with the protective effect of subsequent infection does not necessarily mean no beneficial effect against the subsequent SARS-CoV-2 infection. In contrast, we can infer the potential benefits of bivalent vaccination against SARS-CoV-2 infection based on the observed increase in antibody titer following bivalent vaccination and the correlation between high antibody titer and protection against subsequent SARS-CoV-2 infection.

**STUDY LIMITATIONS**

There are some limitations to this study. First, because all participants who experienced subsequent infections had mild cases of COVID-19, we were unable to determine factors that could protect against severe COVID-19 or death. Also, the predominance of females (especially, nurse) in our study may be a limitation to the generalizability of the results. Additionally, there is a lack of data on T-cell responses or memory immune responses in the study participants. Lastly, as we evaluated the correlates of protection during the BA.5 and BN.1-dominant era, our findings may not be directly applicable to the recent variants like XBB or the upcoming variants. However, we showed that even anti-Wuhan-Hu-1 S1-IgG levels, as well as BA.5 S1-IgG and nAb against BA.5, were immune correlates of protection against SARS-CoV-2 infection. Therefore, we assume that our findings may be valuable for clinical practice and policy decision even in period dominated by new variants.

**Table S1. Incidence of subsequent infections with intervals and sampling intervals.**

|  | **n (%)** |
| --- | --- |
| **All participants** | 482 |
| **Self-reported SARS-CoV-2 infection confirmed by PCR or rapid antigen test** | 53 (10.9) |
| **Asymptomatic infection during the study period defined by serologic test without self-report** | 16 (3.3) |
| Seroconversion of nucleocapsid protein antibody in infection-naïve | 5 (1.0) |
| Two-fold increase of SARS-CoV-2 Wuhan S1-IgG antibody titers in those with previous infection | 11 (2.3) |
| **All subsequent infections including asymptomatic infection during the study period** | 69 (14.3) |
| **Interval between study enrollment and subsequent infection**, days | 35 (13−85) |
| Infection-naive | 34 (13−76) |
| Those with previous infection | 60 (25−95) |
| **Interval between study enrollment and the last blood sampling for serologic test**, days | 104 (92−119) |

Data represent n (%) or median (interquartile range).

**Table S2. Demographics and baseline characteristics of study participants according to subsequent SARS-CoV-2 infection**

| **Variable** | **Total**  **(n=482)** | **Subsequent**  **infection**  **(n=69)** | **No subsequent infection**  **(n=413)** | ***P* value** |
| --- | --- | --- | --- | --- |
| **Age**, years | 33 (28−41) | 35 (28−42) | 33 (28−41) | 0.68 |
| **Male sex** | 74 (15.4) | 13 (18.8) | 61 (14.8) | 0.39 |
| **Underlying comorbidity** | 55 (11.4) | 9 (13.0) | 46 (11.1) | 0.64 |
| **Number of previous vaccinations** |  |  |  | 0.77 |
| 3-dose | 457 (94.8) | 65 (94.2) | 392 (94.9) |  |
| 4-dose | 25 (5.2) | 4 (5.8) | 21 (5.1) |  |
| **Type of previous vaccination** |  |  |  | 0.66 |
| Heterologous | 400 (83.0) | 56 (81.2) | 344 (83.3) |  |
| Homologous | 82 (17.0) | 13 (18.8) | 69 (16.7) |  |
| **Time from last vaccination to study enrollment**, days | 375 (365−383) | 376 (366, 383) | 375 (365, 383) | 0.96 |
| **Previous SARS-CoV-2 infection** | 381 (79.0) | 31 (44.9) | 350 (84.7) | <0.001 |
| **Time from the previous infection**, days^a^ | 251 (127−275) | 269 (116−286) | 250 (127−272) | 0.20 |
| **Bivalent booster vaccination** | 166 (34.4) | 23 (33.3) | 143 (34.6) | 0.83 |
| **Time from last SARS-CoV-2 antigen exposure,** days^a^ | 260 (128−296) | 338 (269−378) | 254 (127−284) | <0.001 |

Data represent n (%) or median (interquartile range).

^a^Calculated without 34 individuals whose infection date was not identified.

**Table S3. Cox-proportional hazard model for the 1-month subgroup analysis for prediction of subsequent infection**

|  | **Univariate analysis** | | **Multivariable analysis** | |
| --- | --- | --- | --- | --- |
| **Variable (prediction by Wuhan S1-IgG)**  **(n=454**^a^**)** | **Hazard ratio**  **(95% confidence interval)** | ***P* value** | **Adjusted hazard ratio**  **(95% confidence interval)** | ***P* value** |
| Homologous previous vaccination | 1.39 (0.66−2.91) | 0.38 |  |  |
| Hybrid immunity | 0.14 (0.08−0.27) | <0.001 | 0.17 (0.09−0.31) | <0.001 |
| Bivalent BA.4/5 booster vaccination | 0.76 (0.38−1.53) | 0.44 |  |  |
| Time since last exposure to SARS-CoV-2 antigen^b^ before study enroll (≤ 90 days) | 0.20 (0.03−1.49) | 0.12 |  |  |
| Wuhan S1-specific IgG ≥1.85^c^ | 0.31 (0.16−0.59) | <0.001 | 0.40 (0.21−0.78) | 0.007 |
| **Variable (prediction by BA.5 S1-IgG)**  **(n=454**^a^**)** |  |  |  |  |
| Homologous previous vaccination | 1.39 (0.66−2.91) | 0.38 |  |  |
| Hybrid immunity | 0.14 (0.08−0.27) | <0.001 | 0.19 (0.10−0.36) | <0.001 |
| Bivalent BA.4/5 booster vaccination | 0.76 (0.38−1.53) | 0.44 |  |  |
| Time since last exposure to SARS-CoV-2 antigen^b^ before study enroll (≤ 90 days) | 0.20 (0.03−1.49) | 0.12 |  |  |
| BA.5 S1-specific IgG level ≥1.55^c^ | 0.21 (0.11−0.41) | <0.001 | 0.30 (0.15−0.59) | <0.001 |
| **Variable (prediction by neutralizing antibody)**  **(n=200**^a^**)** |  |  |  |  |
| Homologous previous vaccination | 1.39 (0.66−2.91) | 0.38 |  |  |
| Hybrid immunity | 0.14 (0.08−0.27) | <0.001 | 0.22 (0.07−0.72) | 0.01 |
| Bivalent BA.4/5 booster vaccination | 0.76 (0.38−1.53) | 0.44 |  |  |
| Time since last exposure to SARS-CoV-2 antigen^b^ before study enroll (≤ 90 days) | 0.20 (0.03−1.49) | 0.12 |  |  |
| Neutralizing antibody against BA.5≥7.63^c^ | 0.09 (0.02−0.39) | 0.001 | 0.15 (0.03−0.75) | 0.02 |

^a^The 1-month subgroup analysis excluded those with the occurrence of subsequent infection within 1 month after bivalent booster vaccination.

^b^Either vaccination or infection

^c^Optimal cutoff value determined from Youden index (values are presented as the log_10_ of the OD ratio for IgG and log_2_ of the ND50 for neutralizing antibody). Antibody titers were used as the baseline antibody level for individuals who did not receive the bivalent vaccine and as the antibody level one month after vaccination for those who did receive the bivalent vaccine.

**Table S4. Demographics and baseline characteristics of study participants according to the bivalent vaccine administration**

| **Variable** | **Bivalent vaccine**  **(n=166)** | **No bivalent vaccine**  **(n=316)** | ***P* value** |
| --- | --- | --- | --- |
| **Age**, years | 39 (32−46) | 31 (27−38) | <0.001 |
| **Male** | 33 (19.9) | 41 (13.0) | 0.046 |
| **Underlying comorbidity** | 25 (15.1) | 30 (9.5) | 0.07 |
| **Number of previous vaccinations** |  |  | 0.02 |
| 3-dose | 152 (91.6) | 305 (96.5) |  |
| 4-dose | 14 (8.4) | 11 (3.5) |  |
| **Type of previous vaccination** |  |  | 0.41 |
| Heterologous | 141 (84.9) | 259 (82.0) |  |
| Homologous | 25 (15.1) | 57 (18.0) |  |
| **Time from last vaccination to study enrollment**, days | 375 (364−383) | 375 (366−383) | 0.99 |
| **Previous SARS-CoV-2 infection** | 119 (71.7) | 262 (82.9) | 0.004 |
| **Time from the previous infection**, days^a^ | 255 (150−271) | 242 (120−277) | 0.13 |
| **Time from last SARS-CoV-2 antigen exposure,** days^a^ | 264 (149−330) | 256 (125−290) | 0.03 |

Data represent n (%) or median (interquartile range).

^a^Calculated without 34 individuals whose infection date was not identified.

**A.**

**
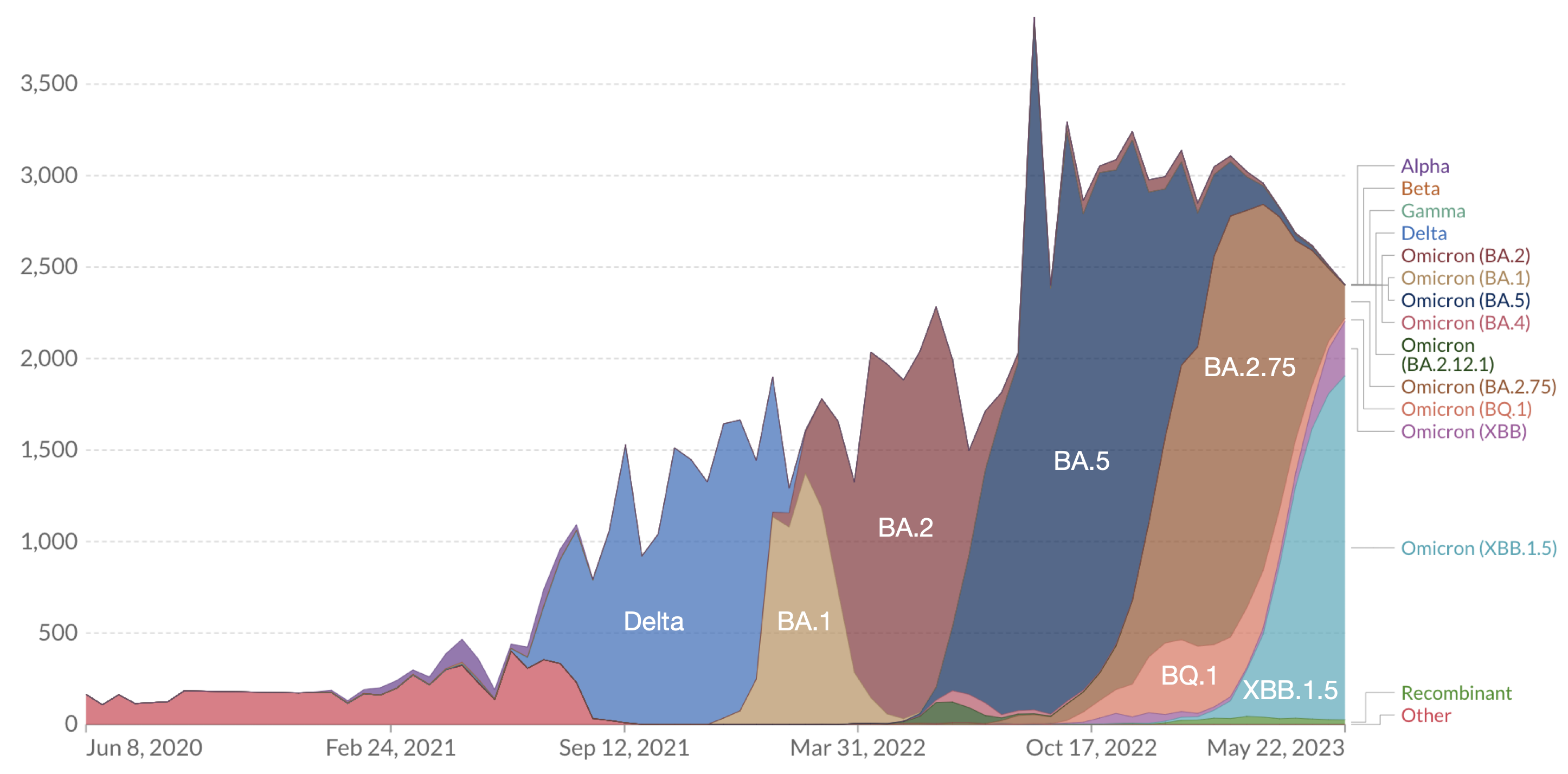
**

**B.**


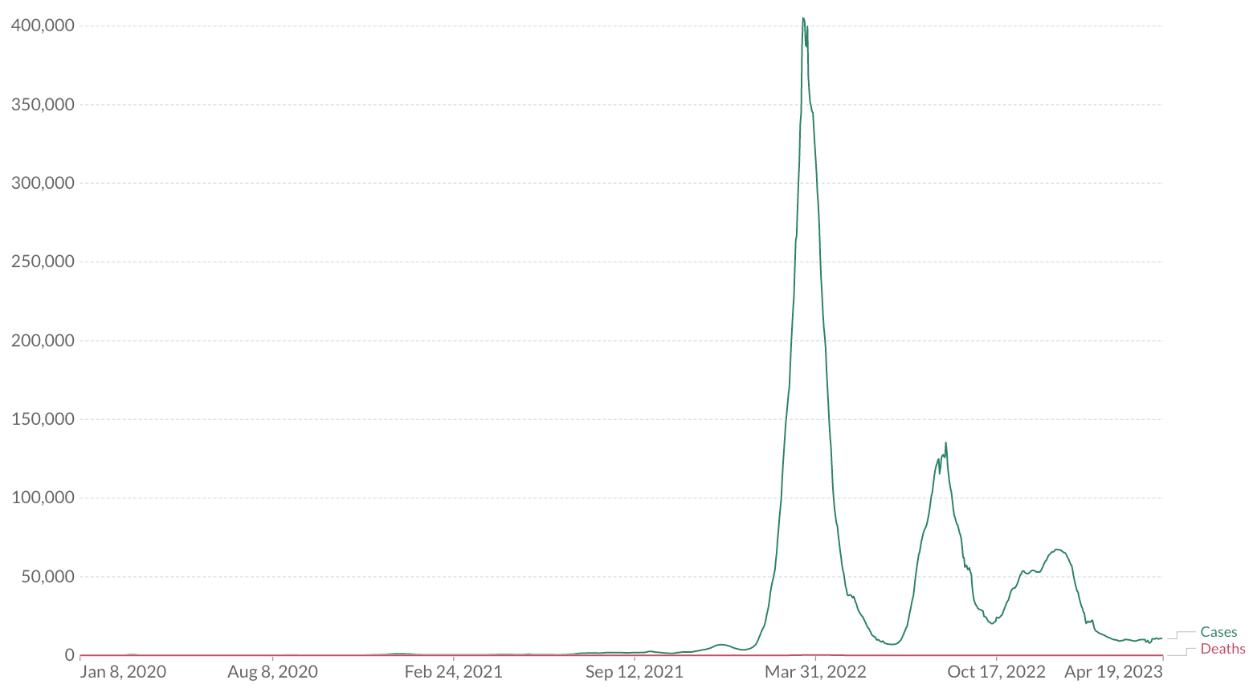


**Figure S1. *A*. SARS-CoV-2 variants in analyzed sequence, and *B*. epidemic curve during the entire period of the COVID-19 pandemic in South Korea**

**
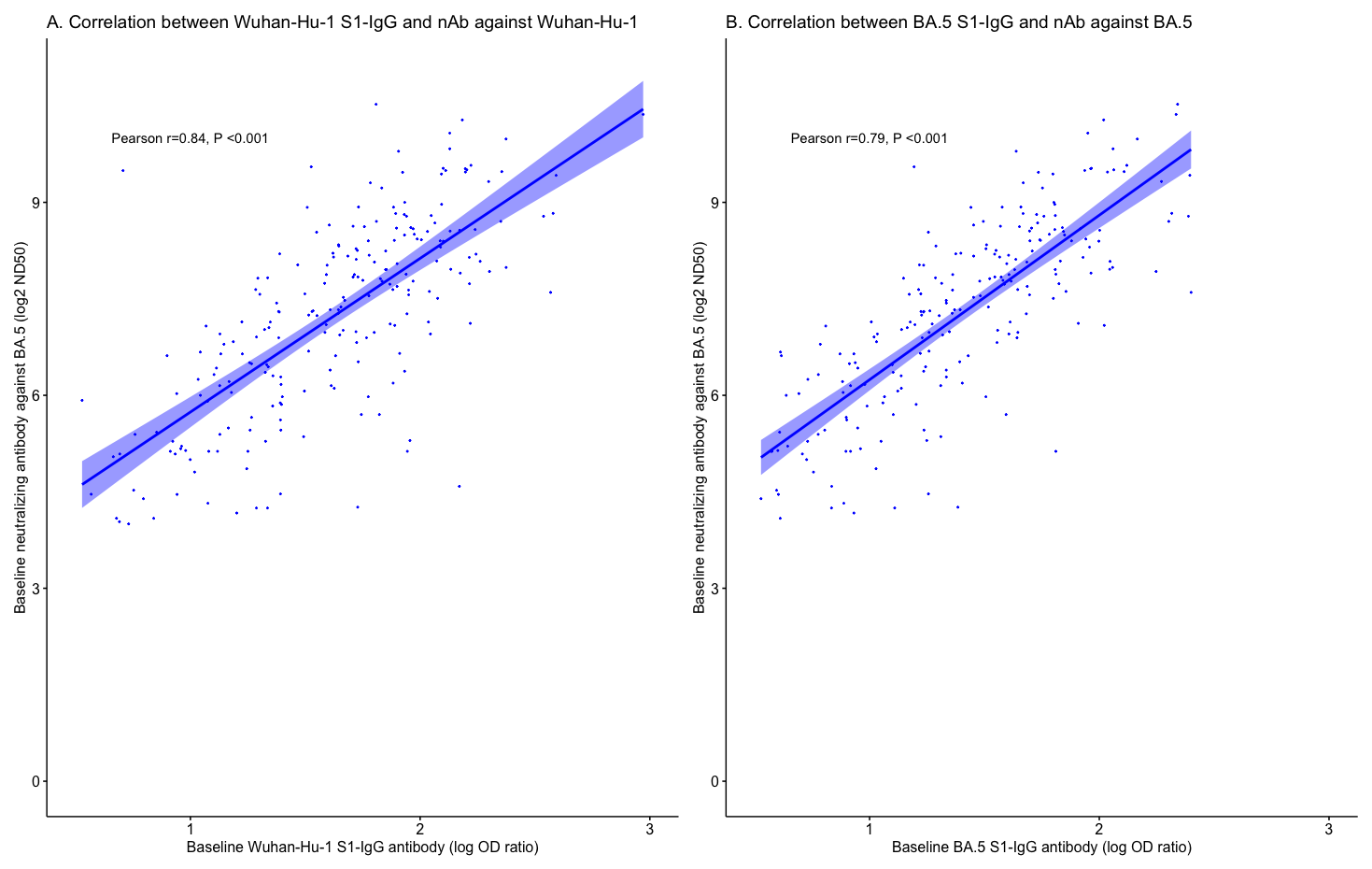
**

**Figure S2. Correlation between serum binding antibody and neutralizing antibody**

**
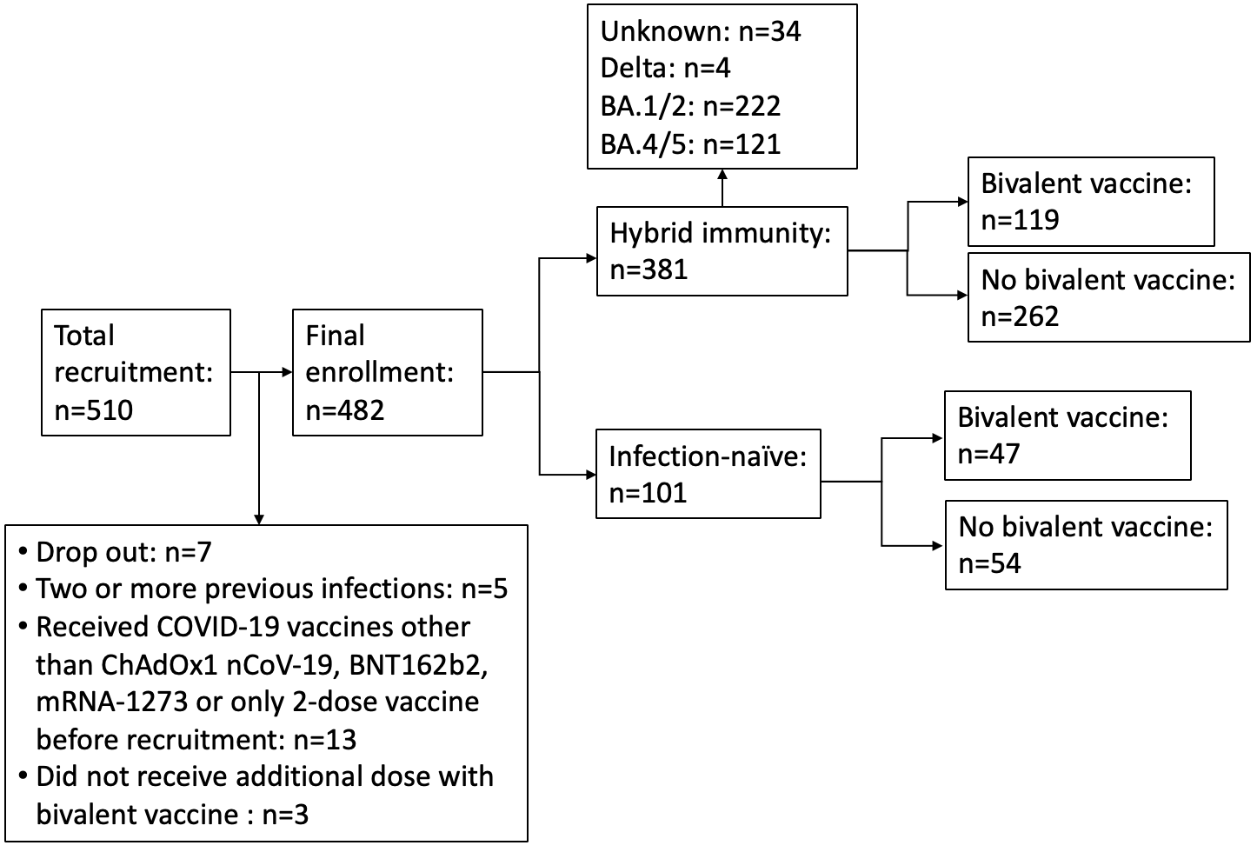
**

**Figure S3. Schematic diagram of the study participants**

**
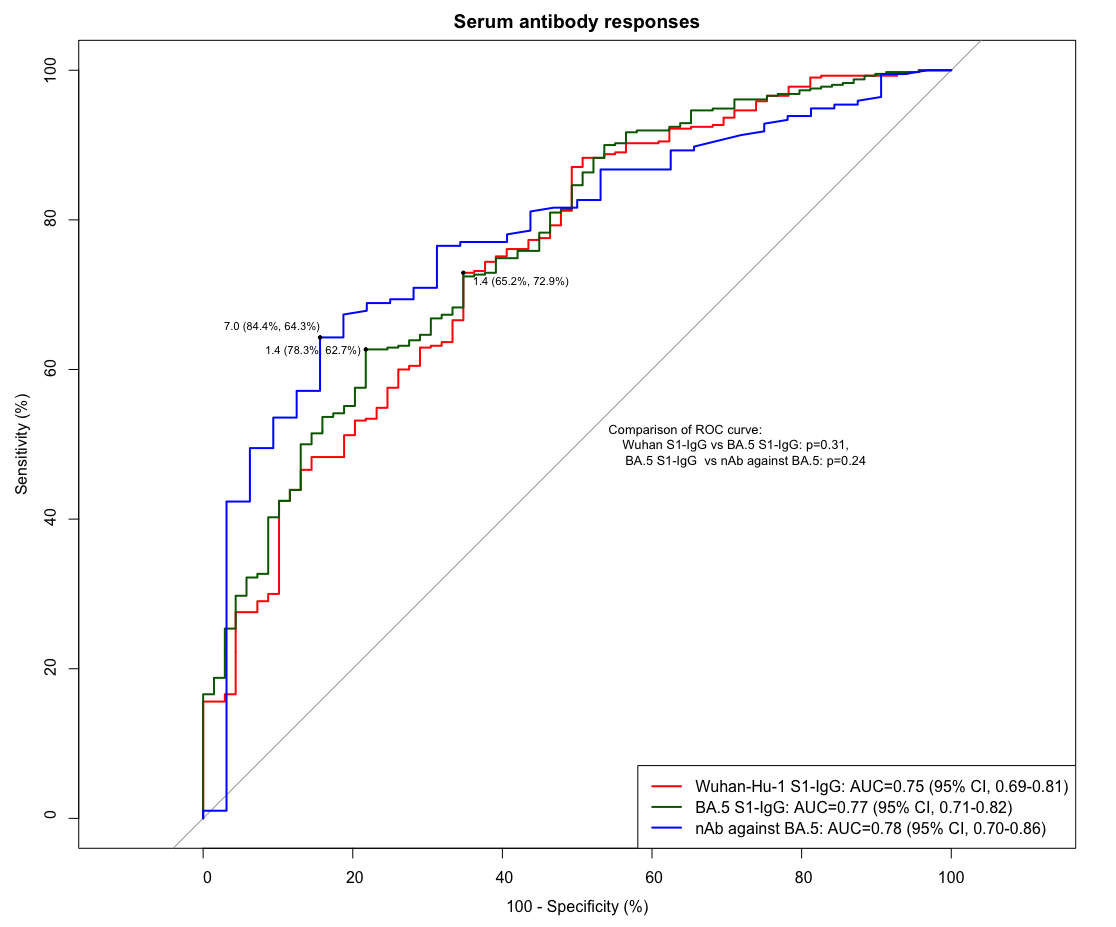
A.**

**
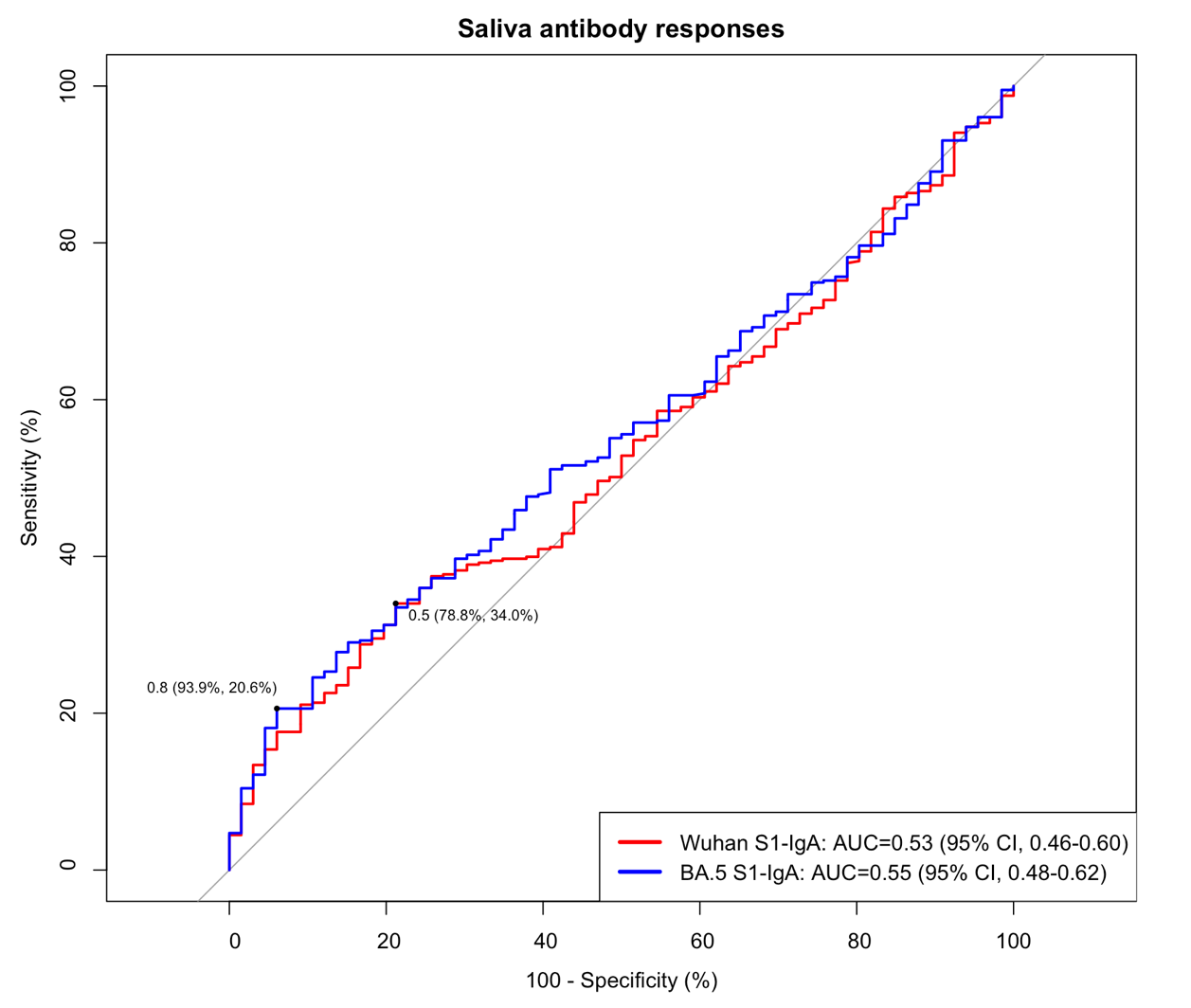
B.**

**Figure S4. Receiver operating characteristic curves of baseline antibody for distinguishing subsequent infection. *A*. serum Wuhan-Hu-1 S1-IgG, BA.5 S1-IgG, and neutralizing antibody against BA. 5. *B*. saliva Wuhan-Hu-1 S1-IgA and BA.5 S1-IgA.** Black dots indicate the optimal cutoff value by the Youden index method, and the two numbers in parentheses refer to specificity and sensitivity, sequentially.


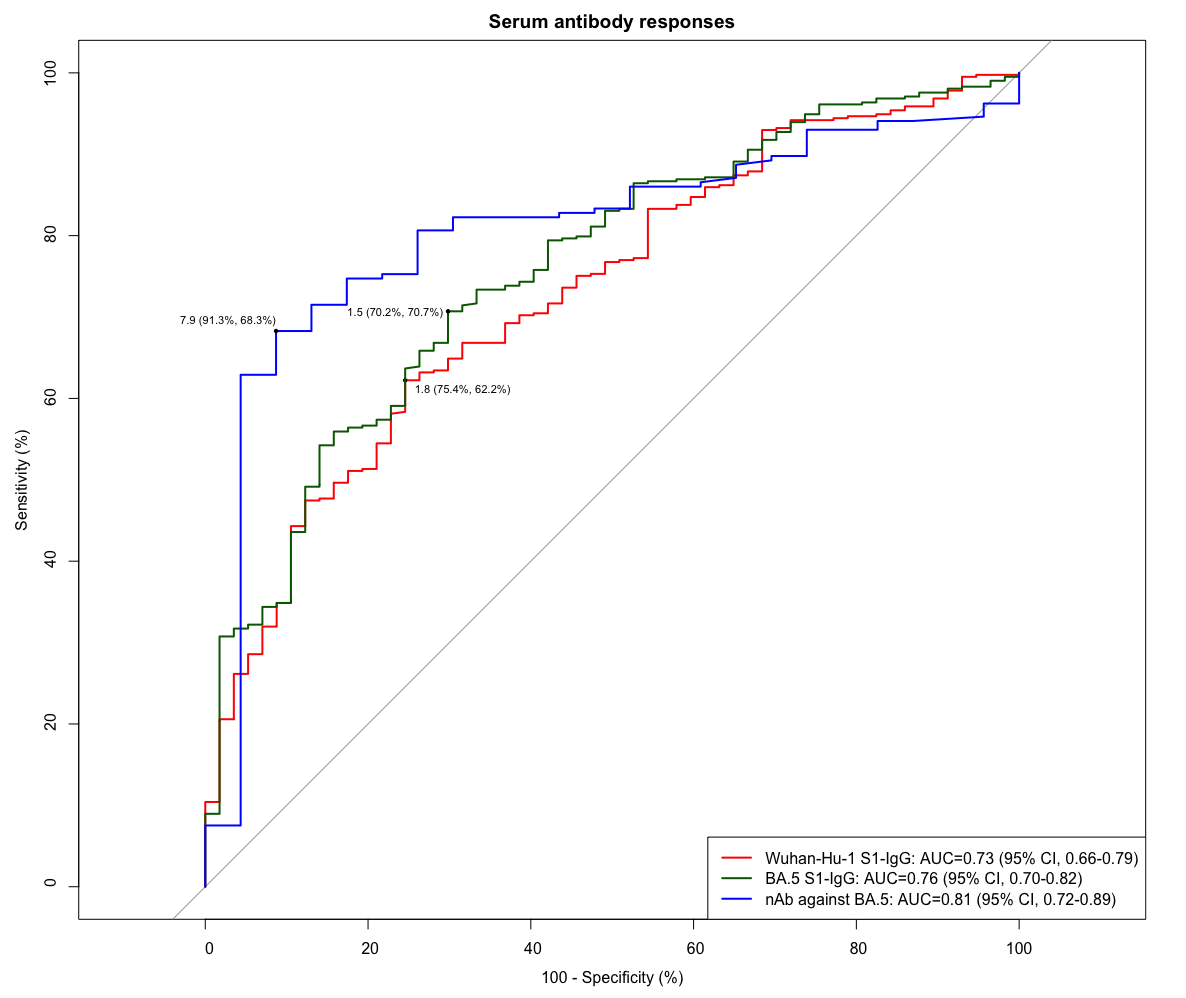


**Figure S5. Receiver operating characteristic curves for 1-month subgroup analysis with serum Wuhan-Hu-1 S1-IgG, BA.5 S1-IgG, and neutralizing antibody against BA.5 for distinguishing subsequent infection.** Black dots indicate the optimal cutoff value by the Youden index method and the two numbers in parentheses refer to specificity and sensitivity, sequentially.


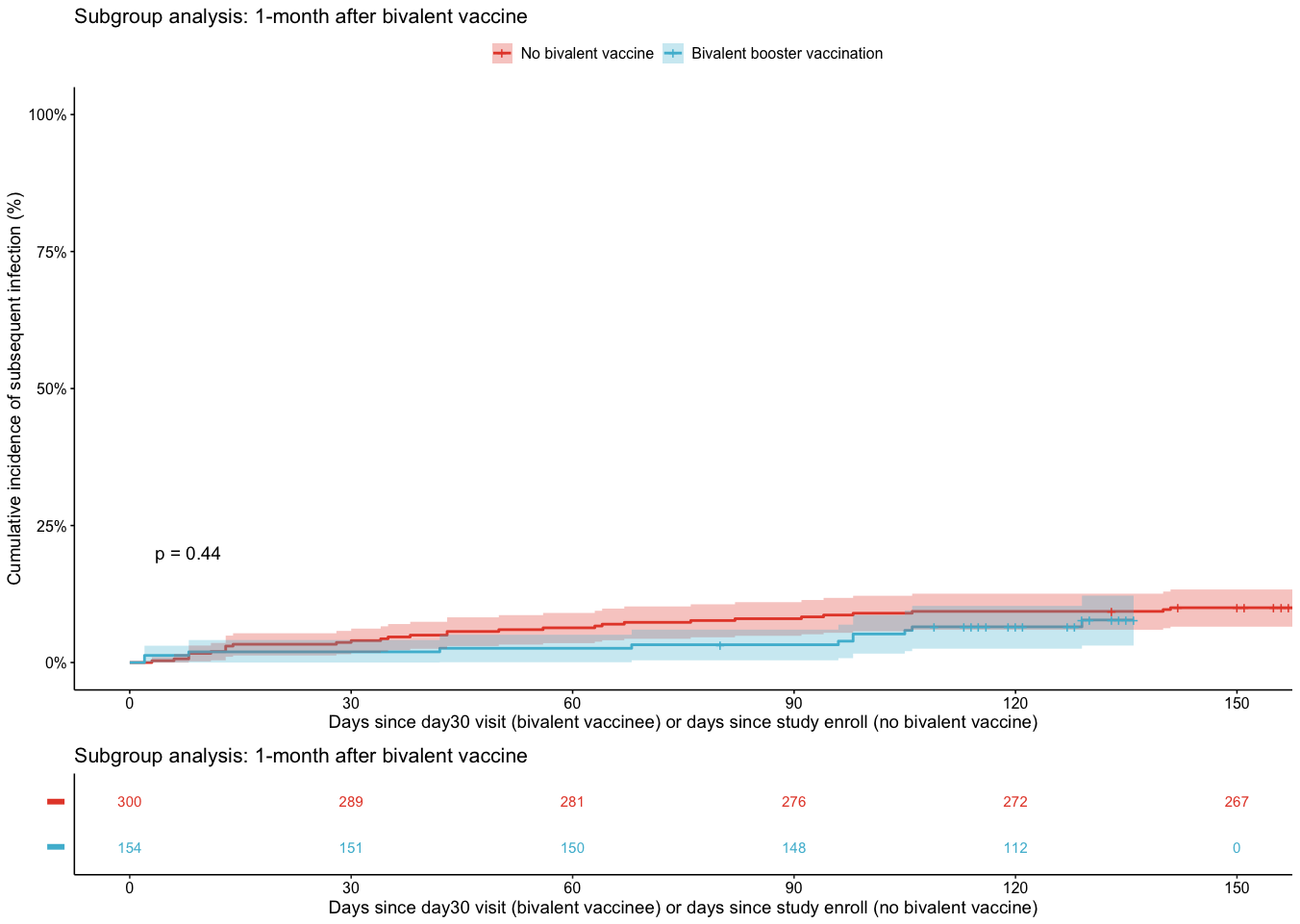


**Figure S6. Cumulative incidence of subsequent SARS-CoV-2 infection according to bivalent vaccine administration in the 1-month subgroup analysis**


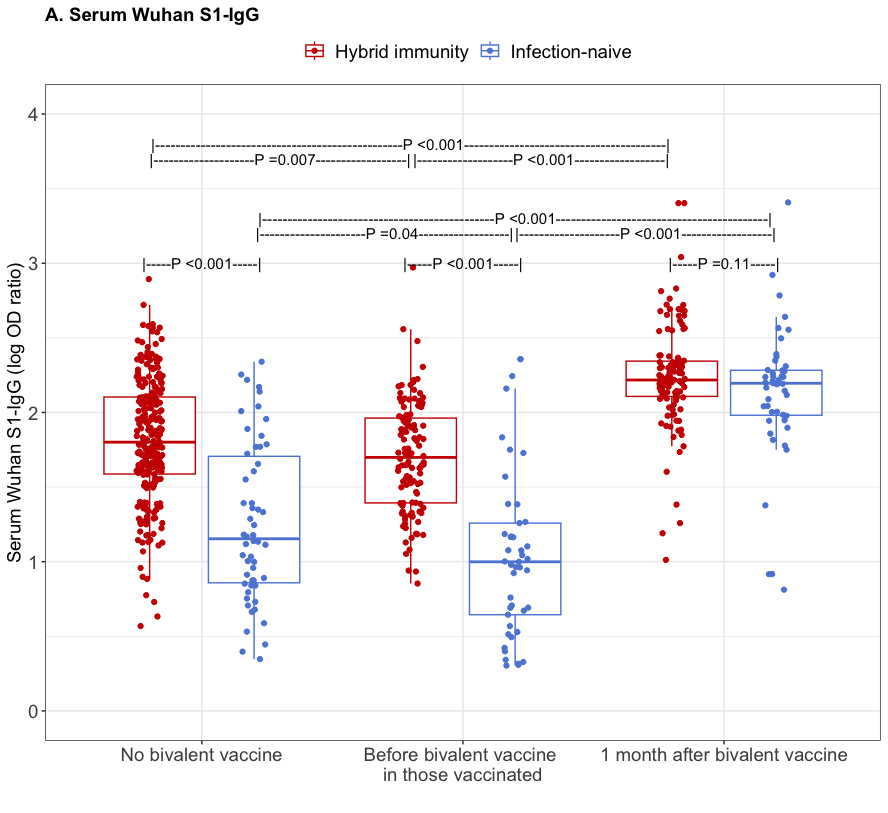

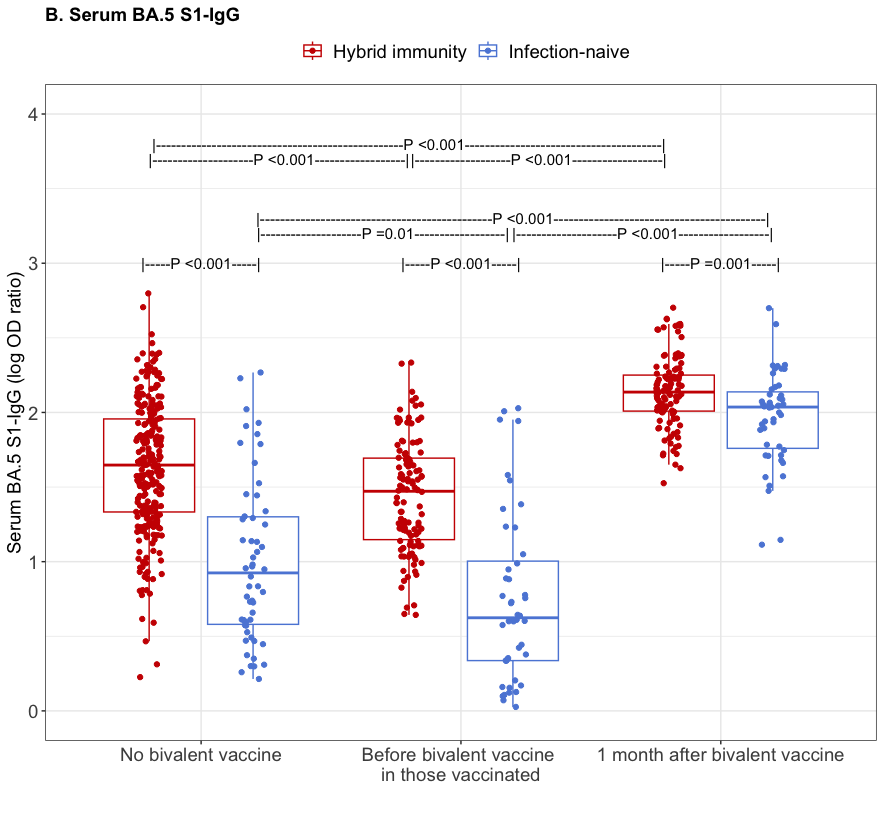

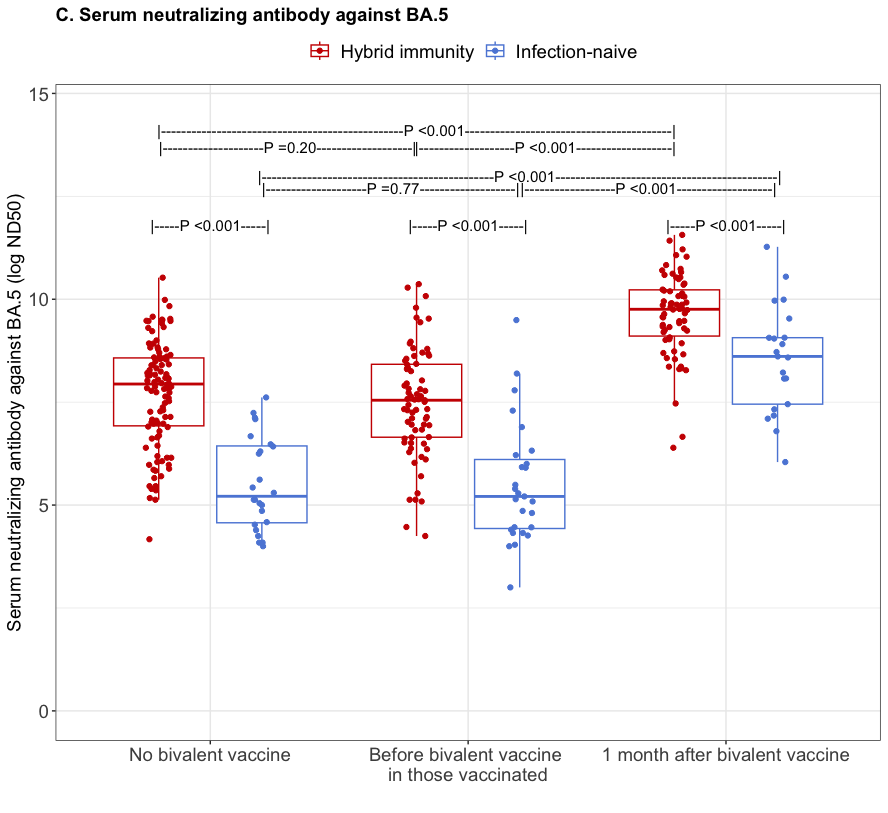


**Figure S7. Serum antibody levels before and after bivalent booster vaccine.** *A*. Serum Wuhan-Hu-1 IgG. *B*. Serum BA.5 S1-IgG. *C*. Serum neutralizing antibody against BA.5. Each box denotes the interquartile range (IQR), where the lower boundary of the box indicates the 25^th^ percentile and the upper boundary indicates the 75^th^ percentile. The line inside the box represents the median value. Whiskers extend from the box to the minimum and maximum values within 1.5 times the IQR.

**REFERENCES**

1. Edouard Mathieu HR, Lucas Rodés-Guirao, Cameron Appel, Charlie Giattino, Joe Hasell, Bobbie Macdonald, Saloni Dattani, Diana Beltekian, Esteban Ortiz-Ospina and Max Roser. "Coronavirus Pandemic (COVID-19)". Available at: https://ourworldindata.org/coronavirus. Accessed May 1.
2. Cauchemez S, Horby P, Fox A, Mai le Q, Thanh le T, Thai PQ, et al. Influenza infection rates, measurement errors and the interpretation of paired serology. PLoS Pathog **2012**; 8(12): e1003061.DOI: http://doi.org/10.1371/journal.ppat.1003061
3. Zhao X, Siegel K, Chen MI, Cook AR. Rethinking thresholds for serological evidence of influenza virus infection. Influenza Other Respir Viruses **2017**; 11(3): 202-10.DOI: http://doi.org/10.1111/irv.12452
4. SARS-CoV-2 variants in analyzed sequences, South Korea. Available at: https://www.kdca.go.kr/contents.es?mid=a20107030000. Accessed April 26.
5. Classen DC, Morningstar JM, Shanley JD. Detection of antibody to murine cytomegalovirus by enzyme-linked immunosorbent and indirect immunofluorescence assays. J Clin Microbiol. 1987 Apr;25(4):600-4. PubMed PMID: 3033015. Pubmed Central PMCID: PMC266042. Epub 1987/04/01.
6. Lardeux F, Torrico G, Aliaga C. Calculation of the ELISA's cut-off based on the change-point analysis method for detection of Trypanosoma cruzi infection in Bolivian dogs in the absence of controls. Mem Inst Oswaldo Cruz. 2016 Jul 4;111(8):501-4. PubMed PMID: 27384081. Pubmed Central PMCID: PMC4981115. Epub 2016/07/08.
7. Pan AA, Rosenberg GB, Hurley MK, et al. Clinical evaluation of an EIA for sensitive and specific detection of serum antibody to Trypanosoma cruzi (Chagas’ disease). *J Infect Dis*. Mar 1992;165(3):585-588.
8. Whiteman MC, Bogardus L, Giacone DG, Rubinstein LJ, Antonello JM, Sun D, et al. Virus Reduction Neutralization Test: A Single-Cell Imaging High-Throughput Virus Neutralization Assay for Dengue. Am J Trop Med Hyg **2018**; 99(6): 1430-9.DOI: http://doi.org/10.4269/ajtmh.17-0948
9. Schisterman EF, Perkins NJ, Liu A, Bondell H. Optimal cut-point and its corresponding Youden Index to discriminate individuals using pooled blood samples. Epidemiology **2005**; 16(1): 73-81.DOI: <http://doi.org/10.1097/01.ede.0000147512.81966.ba>
